# Evaluation of genetic variation in tumor suppressor miRNA encoding and their target genes in breast cancer; focus on miRNA interaction and expression analysis

**DOI:** 10.3389/fgeed.2026.1705463

**Published:** 2026-02-27

**Authors:** Yogita Chhichholiya, Sandeep Singh, Rajesh Vashistha, Manjit Kaur Rana, Anjana Munshi

**Affiliations:** 1 Department of Human Genetics and Molecular Medicine, Central University of Punjab Bathinda, Bathinda, Punjab, India; 2 Department of Oncology, Max Super Speciality Hospital, Bathinda, India; 3 Department of Pathology, All India Institute of Medical Sciences Bathinda, Bathinda, Punjab, India

**Keywords:** 3′UTR, breast cancer, hsa-let-7c, hsa-miR-181c, KRAS

## Abstract

**Background:**

Genetic variations in tumor suppressor miRNAs and the 3′UTR of their target genes influence tumor biology and breast cancer (BC) risk.

**Objective:**

This study investigated genetic variations in tumor suppressor miRNAs (hsa-let-7c, hsa-miR-34a, hsa-miR-145a) and their target genes (KRAS, IGFBP6, IGF1R), and their functional significance in BC patients.

**Methods:**

The miRNA encoding regions and 3′UTRs of the selected target genes were sequenced in 208 BC patients. Functional analyses were performed using luciferase assay, RT-PCR, IHC, and Western blotting. RNAfold, TNM plot, Kaplan-Meier Plotter, and ROC Plotter were used for structural predictions, survival, and therapy response analysis.

**Results:**

Two variants, rs712 and rs9266, were found in the 3′UTR of KRAS. Luciferase assay confirmed that rs9266 disrupts the binding of hsa-let-7c and hsa-miR-181c, leading to increased KRAS expression. KRAS expression was highest in heterozygous, followed by homozygous mutant, and lowest in wild-type genotypes. Higher hsa-let-7c and hsa-miR-181c expression correlated with better survival. ROC analysis identified KRAS as a potential predictive biomarker for chemotherapy response.

**Conclusion:**

Variants rs712 and rs9266 in the KRAS 3′UTR impair miRNA binding, enhancing KRAS expression and tumorigenesis, while elevated hsa-let-7c and hsa-miR-181c levels predict favourable survival outcomes in BC patients.

## Introduction

Despite significant advancements in treatment modalities of BC, the complex molecular mechanisms involved in the progression and resistance to therapy are still being explored (Yeole, 2008). In addition to many other risk factors, epigenetic factors histone modifications, DNA methylation, and non-coding RNAs emerged as potential contributors to the progression and development of the disease. Among the non-coding RNAs, microRNAs (miRNAs) have been explored at length in association with various cancers including BC (Byler et al., 2014). The complementary base-pairing between the seed sequence of miRNA and the 3′UTR of mRNA helps it to regulate the expression of the target gene (Otmani et al., 2022). Because of the short miRNA-mRNA binding site, a single miRNA may attach to numerous targeted mRNAs. Multiple miRNAs can simultaneously target and bind to the same mRNA (Otmani et al., 2022; Bartel, 2009; Melo and Esteller, 2011).

miRNAs are classified as tumor suppressor miRNAs (tsmiRs) or oncogenic miRNAs (oncomiRs). OncomiRs are typically overexpressed in BC, inhibiting the expression of putative tumor suppressor genes. In contrast, tsmiRs suppress the production of oncogenes that promote BC. Variations in miRNA encoding genes as well as in the 3′UTRs of their target genes are emerging as significant players in BC pathogenesis since such variations might prevent miRNA binding, thereby disrupting gene regulation (Vohra et al., 2020; Mulrane et al., 2013).

The present study has been carried out to evaluate variation in tumor suppressor miRNA encoding genes *hsa-let-7c, hsa-miR-34a*, and *hsa-miR-145a* and the 3′UTR of their target genes *KRAS, IGFBP6* and *IGFIR* respectively. Furthermore, the study also aimed to elucidate the functional implications of significant variants as well as their impact on RNA secondary structure and their impact on the disease outcome and response to chemotherapy.

## Materials and methods

Study Population: A cohort of 208 histopathologically confirmed female breast cancer (BC) patients, aged 20–85 years (mean age 55.01 ± 11.9 years), was recruited from Max Super Speciality Hospital and AIIMS, Bathinda, Punjab, India. Additionally, 13 BC tissue biopsy samples were procured from AIIMS Bathinda for functional assays. Participants represented diverse districts across the Malwa region of Punjab. The study protocol received approval from the Institutional Ethics Committee of the Central University of Punjab (CUPB/IEC/1/12/20_10 and CUPB/IEC/2024/22). Comprehensive clinical and demographic data were systematically collected via structured questionnaires, with written informed consent obtained from all patients. Peripheral blood (5 ml) was collected into EDTA vacutainers by trained phlebotomists for genomic DNA isolation.

Genotyping: An extensive literature review utilizing Google Scholar, PubMed, and Scopus identified key tumor suppressor miRNA-encoding genes and their validated targets for this study. Complete sequences of three critical miRNAs, hsa-let-7c, hsa-miR-34a, and hsa-miR-145a and the 3′ untranslated regions (3′UTRs) of their corresponding target genes KRAS, IGF1R, and IGFBP6 were selected for genotypic analysis.

Genomic DNA was extracted from peripheral blood samples using phenol-chloroform method. The full-length sequences of the selected miRNA genes were amplified by PCR and sequenced via Sanger technology. Similarly, 3′UTR regions of KRAS, IGF1R, and IGFBP6 were PCR-amplified using primers designed with a primer design tool (Supplementary Data S1) and subjected to Sanger Sequencing. The genotypic distribution and allelic frequencies were calculated.

Luciferase Reporter Assay: Sanger sequencing revealed two variants—rs712 and rs9266—within the 3′UTR of KRAS; no variants were detected in the miRNA genes or other target genes. To elucidate the functional consequences of rs9266 on miRNA binding, wild-type (WT) and mutant KRAS 3′UTR sequences were cloned into pMIR reporter vectors. The specific sequences for WT and mutant clones are as follows:

Wild type KRAS sequence bearing SNP, rs9266 (T allele)

5′ggc​cgc​AGG​CAT​CAT​GTC​CTA​TAG​TTT​GTC​ATC​CCT​GAT​GAA​TGT​AAA​GTT​ACA​CTG​TT

3′ttc​gaT​CCG​TAG​TAC​AGG​ATA​TCA​AAC​AGT​AGG​GAC​TAC​TTA​CAT​TTC​AAT​GTG​ACA​A

and mutant KRAS sequence bearing SNP, rs9266 (C allele)

5′ggc​cgc​AGG​CAT​CAT​GTC​CTA​TAG​TTT​GTC​ATC​CCT​GAC​GAA​TGT​AAA​GTT​ACA​CTG​TT

3′ttc​gaT​CCG​TAG​TAC​AGG​ATA​TCA​AAC​AGT​AGG​GAC​TGC​TTA​CAT​TTC​AAT​GTG​ACA​A

Approximately, 4*10^4^ MCF-7 cells were seeded in 12 well plates and after co-transfected at 60%–70% confluency with WT or mutant KRAS constructs (100 ng/well) alongside hsa-let-7c (CUG​UAC​AAC​CUU​CUA​GCU​UUC​C) and hsa-miR-181c (AAC​AUU​CAA​CCU​GUC​GGU​GAG) mimics (10 nM/well). Luciferase activity was quantified 48 h post-transfection using Promega Glo Max following lysis and addition of luciferin-containing buffer, assessing the variant’s impact on miRNA-mediated post-transcriptional regulation. All luciferase reporter assays were carried out in biological triplicates. One-way ANOVA was used to analyze the results.

Expression Analysis: KRAS expression was quantified in RNA extracted from 13 BC biopsy samples, stratified by rs712 and rs9266 genotypes: 2 homozygous wild-type (WT), 9 heterozygous (HT), and 2 homozygous mutants (HM). Additionally, expression of the miRNAs hsa-let-7c and hsa-miR-181c, known to bind KRAS 3′UTR, was assessed. β-actin and U6 snRNA served as endogenous controls for mRNA and miRNA, respectively. Relative expression levels were calculated using the 2^−ΔΔCt method. cDNA synthesis followed manufacturer protocols (Bio-Rad), detailed in Supplementary Data S1, including the specific primers for miRNA enrichment. Real-time PCR primer sequences, reaction mixtures, and thermal cycling conditions are provided in Supplementary Data S1. The sequences of the primers used for real-time PCR, the reaction mixture and the conditions for RT-PCR are provided in Supplementary Data S1.

Western blotting: Protein extracts from BC biopsies representing all three KRAS genotypes (rs712 and rs9266; WT, HT, HM) were analyzed via Western blot, using β-actin as the loading control.

### Tissue lysate preparation

Frozen BC tissues were thawed on ice, minced using surgical blades on a chilled glass plate, washed briefly with cold 1X PBS to remove residual blood, and transferred to microcentrifuge tubes. Tissue homogenization was performed in RIPA lysis buffer supplemented with 1% protease inhibitor cocktail to prevent degradation. Samples were further disrupted by ultrasonic probe sonication and vortexed intermittently for 30 min on ice. Lysates were centrifuged at 12,000 rpm for 15 min at 4 °C, and supernatants were collected for protein quantification.

### Quantification of proteins in the lysate

The protein concentration in the lysate was determined with Bradford reagent (Bio-Rad). The protein and the Bradford reagent formed a complex, causing a shift in the absorbance from 465 to 595 nm. The reaction temperature was set at room temperature and 797 μl of Phosphate Buffer Saline (PBS) was added to each reaction tube before adding 200 μl Bradford reagent. 3 μl protein lysate was added to each reaction tube and the blank tube was filled with 3 μl distilled water. A visible color change was observed in the reaction, and optical density was measured in duplicate at 595 nm at 37 °C. The sample’s protein concentration was calculated (μg/μl) using BSA as a standard. To initiate the SDS-PAGE process, 40 μg of each denatured protein sample was loaded in the wells along with a protein marker (Bio-Rad) to determine the molecular weight of the proteins. Resolved proteins were transferred to the nitrocellulose membrane by electro-blotting in transfer buffer 1X for 3 h at 120 mA current. Before initiating the protocol, transfer buffer 1X. With a protein marker, specified molecular weight proteins on the membrane were located, and this section of the membrane was sliced and blocked for 30 min in a 3% BSA or non-fat milk solution in TBST. After blocking, the membrane was washed in TBST (0.02 M Tris HCl, 0.2 M NaCl, and Tween-20- 0,1%) buffer and incubated with a primary antibody made from 3% milk in TBST for 3 h at room temperature. The primary antibodies used were KRAS (1:1,000, Thermo) of 21KD size and b-actin (1:000, CST) of 42 KD size. After 3 h of incubation, the membrane was washed three times in TBST for 10 min each time and incubated in a secondary antibody for the next 2 h. After secondary antibody incubation, the membrane was washed three times in TBST and incubated membrane for 5 min in ECL reagent (Bio-Rad). Immunological detection and imaging were performed by chemiluminescence (Chemi-Doc, Bio-Rad).

Immunohistochemistry: Immunohistochemistry (IHC) was performed using the PolyExcel HRP/DAB detection system (Cat no. PEH002, PathnSitu Biotechnologies Pvt. Ltd.). Formalin-fixed paraffin-embedded breast tissue sections (4 μm) were baked at 70 °C for 20 min, deparaffinized in xylene, rehydrated through graded ethanol series (100%–50%), and rinsed with distilled water. Antigen retrieval was achieved by heat-induced epitope retrieval in EDTA buffer (pH 8.0). Endogenous peroxidase activity was quenched with hydrogen peroxide for 10 min.

Slides were incubated with primary KRAS antibody (1:50) for 30 min, washed with immuno-wash buffer (Cat no. PS006), then incubated with HRP polymer and DAB chromogen. Hematoxylin counterstaining was applied, followed by mounting with DPX and graded ethanol dehydration. A pathologist performed semi-quantitative scoring assessing staining intensity (0 = none, 1 = weak, 2 = intermediate, 3 = strong) and percentage of positive cells (<1% = 0, 1%–10% = 1, 11%–50% = 2, 51%–75% = 3, 76%–90% = 4, >90% = 5) to calculate the histoscore (H-score = Intensity + Proportion).

Statistical Analysis: Data from luciferase reporter assays and qRT-PCR were analyzed using GraphPad Prism 6. Results are expressed as mean ± SEM and evaluated by one-way ANOVA.

Bioinformatic analysis: In order to evaluate structural consequences of rs712 and rs9266 within KRAS 3′UTR, RNA secondary structure predictions were conducted using the RNAfold web server, analyzing both WT and variant sequences.

RNA-Seq Data analysis: The prognostic relevance of KRAS, hsa-let-7c, and hsa-miR-181c expression in BC was examined via Kaplan-Meier survival analysis. Clinical datasets with expression data were extracted from GEO and consolidated into the Kaplan-Meier plotter platform (Bray et al., 2018). Survival outcomes analyzed included Distant Metastasis-Free Survival (DMFS), Overall Survival (OS), Progression-Free Survival (PPS), and Relapse-Free Survival (RFS), leveraging RNA-chip data. miRNA expression association with OS was analyzed using RNA-Seq data.

The predictive value of KRAS expression for chemotherapy response in BC patients with grade 2 and 3 tumors was evaluated using ROCplot.org, incorporating GEO platform datasets (“GPL96,” “GPL570,” and “GPL571”) with search terms “breast,” “cancer,” and “therapy” (Jirtle and Skinner, 2007). ROC curves were generated, with Area Under the Curve (AUC) quantifying predictive accuracy. This analysis measured KRAS expression’s ability to discriminate responders from non-responders based on sensitivity and specificity.

## Results

### Demographic characteristics

The comprehensive clinical and demographic characteristics of the 208 breast cancer patients are presented in Supplementary Data S1, providing a robust cohort for genetic and functional analyses.

### Genotypic distribution

The Sequencing analysis revealed the exclusive presence of two critical variants, rs712 and rs9266, within the KRAS 3′UTR region. The genotypic distribution wild-type (WT), heterozygous (HT), and homozygous mutant (HM) was well balanced, underscoring the relevance of these variants in this patient population (Supplementary Data S1).

In the current study. the proportion of patients with wild type TT/TT genotype of the two variant was slightly lower in the younger group of patients 10.16% (diagnosed before 45 years) compared to the older group of patients 13.42% (diagnosis after the age of 45). The frequency of the altered rs712 and rs9266 genotypes (TG + GG/TC + CC) was higher in both groups. It was observed to be 89.83% in the younger group and 86.57% in the older group. In the patients with age at 1st pregnancy ≥25 years group, 15.12% (18/119) carried the wild-type TT/TT genotype, whereas 84.87% (101/119) carried the altered genotypes TG + GG/TC + CC for both the variants rs712 and rs9266. In the <25 years group, 10.71% (9/84) had the TT/TT genotype, and 89.28% (75/84) carried the TG + GG/TC + CC genotype. Pre-menopausal patients (n = 91) showed a 13.18% (12/91) frequency of the wild-type TT/TT genotype. The frequency of the altered genotypes for rs712 and rs9266 i.e., TG + GG/TC + CC was found to be 86.81%. Post-menopausal patients (n = 117) showed 12.82% (15/117) for wild-type TT/TT genotype and 87.17% (102/117) carried altered genotype TG + GG/TC + CC genotypes for both the variant. Tumor grade was also assessed in patients in association with the various genotypes. Patients with Grade 2 (n = 170) and Grade 3 (n = 38). Patients with grade 2 tumor carried the wild-type TT/TT genotype in 12.94% (22/170), and 87.05% (148/170) had the TG + GG/TC + CC genotypes (rs712 and rs9266). In patients with Grade 3 tumor, the distribution was 13.15% (5/38) for the TT/TT genotype and 86.84% (33/38) for the altered TG + GG/TC + CC genotypes.

### Impact of miRNA binding by rs9266 on KRAS expression

Luciferase reporter assays decisively demonstrated that the rs9266 variant profoundly disrupts the binding of tumor suppressor miRNAs to the KRAS 3′UTR, most notably hsa-miR-181c. The presence of hsa-miR-181c led to a significant suppression of luciferase activity in WT constructs, confirming direct targeting of KRAS. Strikingly, the rs9266 mutation abolished this suppression, resulting in dramatically elevated luciferase activity in mutant constructs, indicative of a robust derepression and consequent upregulation of KRAS expression.

**FIGURE 1:**
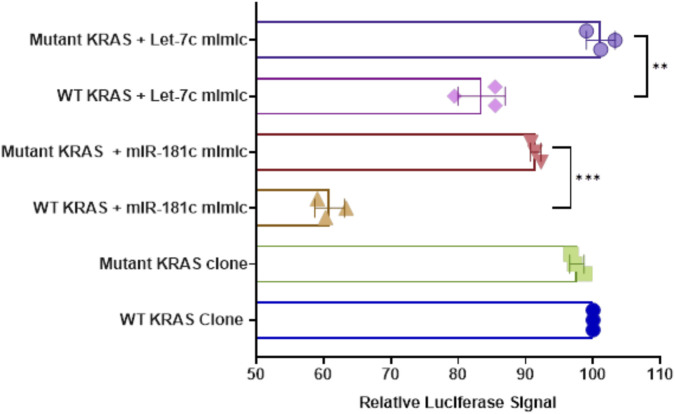
Evaluation of SNP rs9266 within 3′UTR of *KRAS* gene and its effect on binding of *hsa-miR-181c* and *hsa-let-7c* in MCF-7 cells. All luciferase reporter assays were carried out in biological triplicates. One-way ANOVA was used to analyse the results.

While the effect of rs9266 on hsa-let-7c binding was comparatively moderate, it remained statistically significant, revealing that these variant compromises multiple layers of miRNA-mediated regulation of KRAS (****p < 0.0001) (Figure 1). Collectively, these findings establish rs9266 as a critical functional variant that undermines tumor suppressive miRNA control, thereby driving oncogenic KRAS overexpression and potentially fueling breast cancer progression.

### Expression patterns of *hsa-miR-181c* and *hsa-let-7c*

**FIGURE 2:**
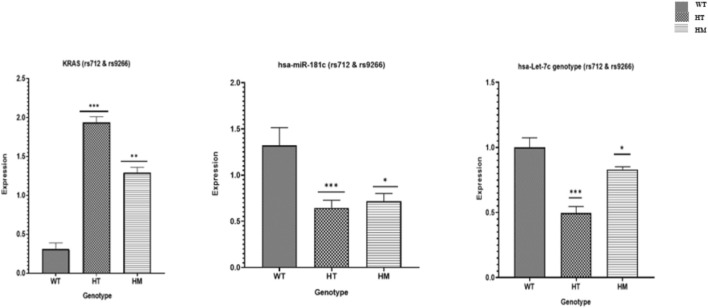
Relative mRNA expression levels of the *KRAS* gene, *hsa-miR-181c* and *hsa-let-7c* in different genotype groups (rs712 and rs9266) i.e., WT, HT and HM. The relative fold change is expressed as 2-^ ^ΔΔCT^.

KRAS expression exhibited a highly significant, genotype-driven escalation across rs712 and rs9266 variants (p < 0.0001), revealing a clear molecular gradient: WT carriers maintained minimal KRAS expression, whereas HT patients displayed a dramatic surge the highest observed followed by a substantial, though slightly lower, increase in HM. This pattern unequivocally demonstrates that these variants exert a powerful modulatory effect on KRAS levels. In contrast, the tumor suppressor miRNAs hsa-miR-181c and hsa-let-7c showed a compelling inverse expression pattern. WT individuals possessed robust miRNA expression, essential for maintaining tight KRAS regulation. However, HT carriers exhibited a significant decline, and HM patients suffered the most severe depletion of these critical miRNAs (p < 0.0001) (Figure 2).

This profound inverse correlation highlights the disruption of a vital regulatory axis whereby the integrity of hsa-miR-181c and hsa-let-7c is compromised by the genetic variants, unleashing KRAS overexpression and fueling oncogenic drive. Together with luciferase assay results, these findings establish a mechanistic link between variant-induced miRNA binding disruption and aggressive KRAS-mediated breast cancer pathogenesis, positioning these miRNAs as pivotal tumor suppressors whose loss indicates poor disease control.

### IHC and Western blot validation of genotype-dependent KRAS expression

**FIGURE 3:**
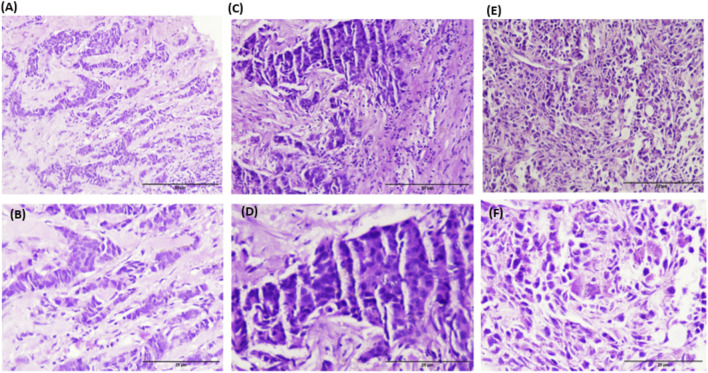
Representative photographs of H and E staining of BC tissues **(A,B)** WT genotype; **(C,D)** HT genotype; **(E,F)** HM genotype.

**FIGURE 4:**
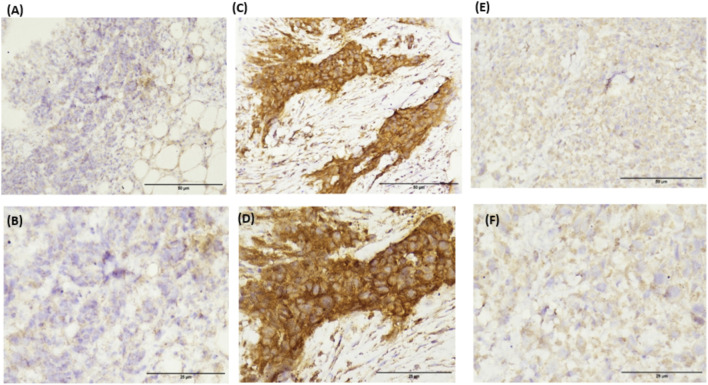
Representative photographs of IHC showing expression of KRAS in the BC patients; **(A,B)** WT genotype; **(C,D)** HT genotype; **(E,F)** HM genotype.

**FIGURE 5:**
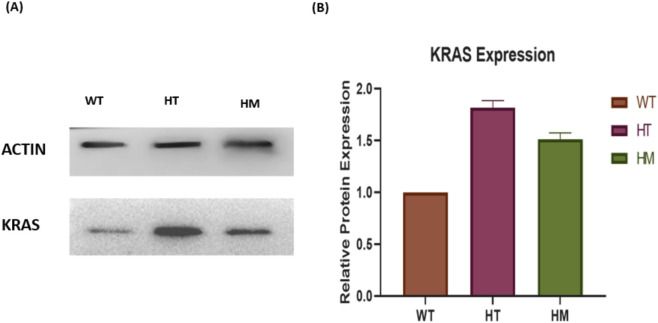
**(A)** Western blot Analysis of *KRAS* in WT genotype rs712 (TT) and rs9266 (TT); HT genotype rs712 (TG) and rs9266 (TC) and HM genotype rs712 (GG) and rs9266 (CC). The control image shown here has not been used before for illustration. **(B)** Densitometric Analysis of protein expression of *KRAS* in WT, HT and HM genotype. Original blot image has been added to the Supplementary Data S2, Figure 7.

Immunohistochemistry (IHC) and Western blot analyses robustly corroborated the genotype-driven modulation of KRAS protein expression. Breast cancer tissues from WT genotype carriers exhibited faint KRAS staining and correspondingly low protein levels, highlighting minimal oncogenic activation. In stark contrast, HT genotypes demonstrated the most intense immunostaining and markedly elevated KRAS protein abundance, reflecting a dramatic upregulation at the translational level. Homozygous mutants (HM) displayed intermediate staining intensity and protein expression, reinforcing the graded impact of these variants on KRAS expression (Figures 3–5).

These protein-level validations powerfully confirm the functional consequences of rs712 and rs9266 variants, linking genotype directly to oncogenic KRAS overexpression in breast tumors.

### RNA secondary structure prediction

To understand the potential structural implications of rs712 and rs9266 in the 3′UTR of *the KRAS* gene, the RNA secondary structure prediction was made using the RNAfold web server (Supplementary Data S2, Figures 1, 2). The sequences provided were the WT and altered sequence bearing rs712 and rs9266.

The optimal secondary structure of the WT 3′UTR of KRAS (rs712 and rs9266) had a Minimum Free Energy (MFE) of −1,336.50 kcal/mol, indicating high structural stability. The thermodynamic ensemble’s free energy was −1,435.82 kcal/mol, with the MFE structure occurring at 0.00%, suggesting multiple viable conformations in a biological environment. The centroid structure had an MFE of −1,014.95 kcal/mol, representing the most probable RNA conformation.

For the altered sequence, the MFE was slightly lower at −1,339.40 kcal/mol, suggesting increased stability. The thermodynamic ensemble’s free energy was −1,438.44 kcal/mol, while the centroid structure had an MFE of −1,127.11 kcal/mol, indicating a structural shift. The ensemble diversity decreased from 1,513.11 (WT) to 1,321.88 (altered), implying reduced variability. These findings highlight the potential structural impact of rs712 and rs9266 on KRAS RNA stability.

### Mountain plot analysis

Mountain plots provide a visual representation of RNA secondary structures based on the sequence of base pairs. These plots help to compare the different RNA conformations, particularly in highlighting structural differences between WT and altered forms. Supplementary Data S2, Figure 2 illustrates the Mountain Plot comparison of the WT and altered *KRAS* sequences. The plot displays three distinct curves representing the MFE structure, the thermodynamic ensemble, and the centroid structure for both sequences.

### Kaplan-Meier plot: *KRAS* expression using RNA-Chip data and patient survival

**FIGURE 6:**
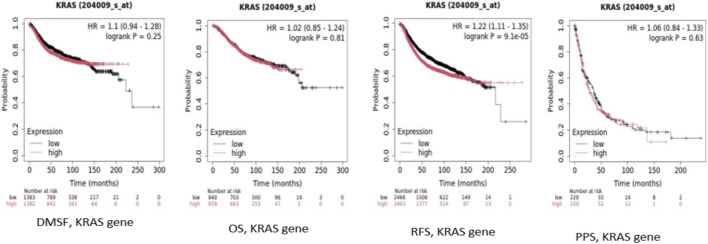
Kaplan-Meier plot of *KRAS* expression using RNA Chip data showing association with survival outcomes across all categories (DMFS, OS, RFS, and PPS).

The Kaplan-Meier survival curves based on RNA chip data, stratified patients into high and low-expression groups as per the expression of *KRAS* (Figure 6). The survival outcomes measured included: DMSF, OS, RFS, and PPS. However, *KRAS* did not show a significant association with DMFS, OS, and PPS (p > 0.05) whereas, RFS showed a significant association between high *KRAS* expression as indicated by the higher HR and the clear separation of the survival curves (p < 0.05).

### *hsa-let-7c* and *hsa-miR-181c* expression analysis using RNA-Seq data

**FIGURE 7:**
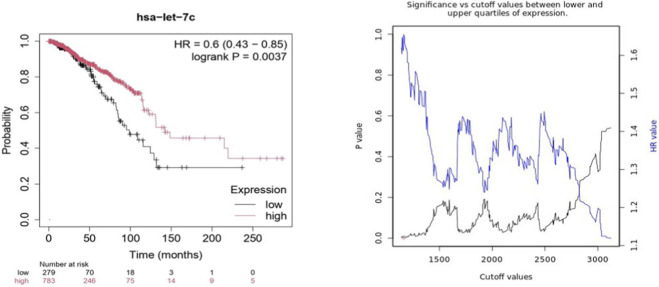
Kaplan-Meier Plot of *hsa-let-7c* expression using RNA-Seq data in BC.

Kaplan-Meier survival analyses based on RNA-seq data revealed a clear and significant survival advantage for breast cancer patients with elevated expression of hsa-let-7c (Figure 7). High levels of hsa-let-7c strongly associated with improved overall survival (OS), with patients exhibiting longer survival durations compared to those with low expression. The hazard ratio (HR) was less than 1, signifying a protective effect of hsa-let-7c against mortality. This association was statistically robust, with a log-rank p-value of <0.0037, confirming the tumor-suppressive role of hsa-let-7c in breast cancer.

**FIGURE 8:**
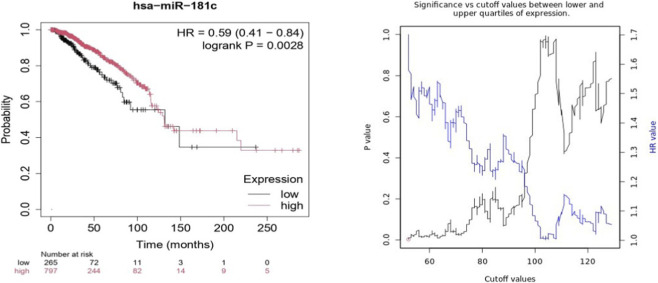
Kaplan-Meier Plot of hsa-miR-181c expression Using RNA-Seq Data in BC.

Similarly, Kaplan-Meier curves for hsa-miR-181c expression (Figure 8) demonstrated that patients with high hsa-miR-181c expression also experienced significantly better OS. The HR indicated a decreased risk of death in this group, reinforcing the notion that hsa-miR-181c acts as a critical protective miRNA in breast cancer. The log-rank p-value of <0.0028 confirms the significance of this survival benefit.

### ROC analysis of *KRAS* expression as a predictive biomarker in BC using RNA-Seq data

Receiver Operating Characteristic (ROC) curve analysis was performed to assess the predictive potential of KRAS expression in breast cancer patients undergoing chemotherapy, stratified by tumor grade. For grade 2 tumors, the ROC curve demonstrated a moderate Area Under the Curve (AUC) of 0.58 with a highly significant p-value (<0.0001) (Supplementary Data S2, Figure 3). This indicates that KRAS expression provides moderate predictive accuracy for chemotherapy response in this subgroup. While the balance of sensitivity and specificity suggests KRAS expression has some value in forecasting therapeutic outcomes, it is not sufficiently robust to serve as a standalone biomarker and should ideally be integrated with other clinical parameters to enhance predictive power.

Conversely, the ROC curve for grade 3 tumors showed no statistically significant predictive capacity (Supplementary Data S2, Figure 4). Despite some indication of predictive accuracy, the lack of significance suggests KRAS expression alone is insufficient to reliably predict chemotherapy response in patients with more aggressive, high-grade tumors.

## Discussion

BC remains the most prevalent and deadly invasive malignancy in women worldwide, demanding urgent advances in understanding its molecular drivers (Bray et al., 2018). While environmental factors contribute, genetic predisposition particularly involving oncogenes and tumor-suppressive miRNAs plays a decisive role in BC pathogenesis (Jirtle and Skinner, 2007). Yet, the role of genetic variants within tumor-suppressive miRNA genes and their target gene 3′UTRs remains largely an underexplored area. Our study added to this by identifying two critical polymorphisms, rs712 and rs9266, within the 3′UTR of the key oncogene KRAS variants that profoundly reshape miRNA-mediated regulation in BC. Despite no detected variants in the miRNAs themselves or in IGFBP6 and IGF1R targets, these KRAS 3′UTR variants emerged as hotspots with consistent presence in BC patients.

Kim et al. (2014) reported the association of KRAS rs712 and rs9266 with the development and prognosis of lung and ovarian malignancies. KRAS is a major oncogene that affects cell proliferation, differentiation, division, and apoptosis. The rs712 and rs9266 have a functional role in controlling KRAS expression since these are present in the 3′UTR which is complementary site for several miRNAs, like let-7 and miR-181 (Kim et al., 2014).

However, Jin et al (2022), did not observe an association between *KRAS* rs712 and BC risk (Jin et al., 2023). These findings were in consensus with a previous study carried out by Huang et al (2015) who also could not establish this association with BC (Huang et al., 2015). On the contrary, Sanaei et al. (2017) observed that the *KRAS* rs712 TT vs. GG + GT genotype is associated with a reduced BC risk in an Iranian population (Sanaei et al., 2017). The fact that the research subjects were from different populations and that sample sizes varied could be reasons for the discrepancies in the findings of various studies. Another study evaluating an association between *KRAS* rs712 and other tumors by meta-analysis found that the risk of colorectal cancer in the East Asian population is increased on account of rs712. At the same time, rs712 was reported to have a protective effect in colorectal cancer in the European population. It has been suggested that this locus might have a different impact in different ethnic groups on account of various parameters used for population stratification (Gholami et al., 2019). Hu et al. (2015) observed that in lung cancer tissues, the homozygous mutant GG (rs712) genotype group had considerably higher levels of KRAS mRNA expression than the patients bearing heterozygous TG and TT (WT). Functional analysis demonstrated that rs712 compromised let-7g binding to the KRAS 3′ UTR, completely abrogating miRNA/mRNA interaction in lung cancer cells. However, they observed similar let-7g (that targets KRAS) expression levels in all the genotypes based on RT-PCR, Western blot and IHC (Hu et al., 2015). Luciferase reporter assays confirmed a ∼15% reduction in KRAS expression for rs712*G and ∼9% for rs9266*C compared to the WT genotype, with ∼30% suppression when both altered alleles were present (Kim et al., 2014).

In the present study, HT (TG/TC) and HM (GG/CC) genotypes for rs712 and rs9266 were more frequent in BC patients than WT (TT/TT), indicating a potential risk association. The luciferase assays in MCF-7 BC cells further revealed that rs9266 significantly disrupted KRAS-hsa-miR-181c interaction, reducing miRNA binding. While rs9266 also impacted hsa-let-7c binding, its effect was less pronounced. These findings suggest that rs9266 contributes to KRAS overexpression by interfering with miRNA-mediated regulation, promoting tumor progression. KRAS expression analysis through RT-PCR, Western blot, and IHC in BC tissue samples showed that the WT genotype (TT for rs712 and rs9266) exhibited the lowest expression levels, while HT (TG for rs712, TC for rs9266) individuals showed the highest expression followed by HM (GG for rs712, CC for rs9266) of KRAS (the results of all these were in consensus). These results indicate that rs712 and rs9266 variants modulate KRAS expression by disrupting miRNA interactions. Elevated KRAS levels lead to tumor proliferation, survival, and invasion via MAPK/ERK and PI3K/AKT pathways (Chhichholiya et al., 2024).

In the current study an inverse correlation was observed between hsa-miR-181c, hsa-let-7c and KRAS expression in the patients bearing different genotypes of two variants rs712 and rs9266 was under focus. WT individuals showed the highest expression levels, lowest expression in HT followed by HM groups. This highlights the tumor-suppressive role of hsa-miR-181c in KRAS regulation. Similar trends have been observed in brain and lung cancers, where miR-181a was reported to limit cell proliferation (Ma et al., 2015; Zhang et al., 2015). In cutaneous squamous cell carcinoma (CSCC), increased miR-181a expression reduced tumor growth by 80% *in vivo*, correlating with reduced KRAS and phospho-ERK levels (Neu et al., 2017). The let-7 family’s tumor-suppressive role is well-established, targeting oncogenes like KRAS to inhibit proliferation and metastasis. The downregulation of hsa-let-7c in altered genotypes supports the hypothesis that rs712 disrupts its binding to KRAS 3′ UTR. Similar findings have been reported in prostate cancer patients bearing rs712 (Chhichholiya et al., 2024).

RNA secondary structure analysis using the RNAfold web server revealed that rs712 and rs9266 induced structural alterations, potentially impacting miRNA binding and RNA-protein interactions. These changes could disrupt regulatory mechanisms in BC cells, contributing to oncogenesis.

Kaplan-Meier analysis using KM plotter confirmed that high KRAS expression correlates with poor outcomes in BC, particularly in OS and RFS as per RNA-chip as well as RNA-seq datasets. The significant association between KRAS and RFS highlights its role in disease recurrence. Elevated KRAS levels may enhance tumor proliferation and survival through MAPK/ERK and PI3K/AKT pathways, increasing recurrence risk. However, KRAS expression did not show a significant association with PFS and DMFS, suggesting it is not the sole determinant of metastatic spread or resistance to various therapeutic strategies. Kaplan-Meier survival analysis of hsa-let-7c and hsa-miR-181c revealed their tumor-suppressive roles, with high expression correlating with improved OS. The protective effect of hsa-let-7c (HR < 1) highlights its importance in the repression of KRAS, consistent with previous studies on the let-7 family (Gilles and Slack, 2018). Similarly, high hsa-miR-181c levels are associated with better OS, reinforcing its role in inhibiting KRAS and its downstream signaling pathways. The significant log-rank p-values for both miRNAs suggest a strong prognostic potential.

ROC analysis highlights KRAS as a predictive biomarker for response in BC chemotherapy, revealing distinct trends between grade 2 and grade 3 tumors. In grade 2 patients, KRAS expression showed moderate predictive value (AUC indicating a balance between sensitivity and specificity), suggesting its potential role in identifying the chemotherapy responders. However, its predictive value in grade 3 patients was limited, likely due to tumor heterogeneity and therapy resistance. These findings emphasize the need for comprehensive predictive models integrating multiple biomarkers and clinical parameters.

Based on the combined Kaplan-Meier survival and ROC analyses, the prognostic and therapeutic significance of KRAS, hsa-let-7c, and hsa-miR-181c in BC has emerged. High KRAS expression is consistently linked to poor outcomes, whereas elevated hsa-let-7c and hsa-miR-181c levels improve survival. Integrating these molecular markers into BC management strategies could facilitate personalized treatment approaches. Future research should validate these findings in larger cohorts and explore targeted therapeutic strategies. KRAS has been extensively studied in other cancers, including lung, colorectal, and pancreatic malignancies (Pan et al., 2019; Abou-Alfa et al., 2011).

KRAS has emerged as a significant contributing factor in BC. However, other variables including age at diagnosis, age at 1^st^ pregnancy, menopausal status and tumor grade have also been implicated in the development of BC. Many other confounding factors such as treatment differences and patient demographics might also be associated with the pathogenesis of BC on account of KRAS dysregulation. However, this needs to be validated in a larger cohort so that a proper conclusion could be inferred.

Recent studies highlight the crucial modulation of oncogene expression and tumor behavior by non-coding RNA networks and transcriptome-level architecture. The biological plausibility that 3′UTR variants that disrupt miRNA binding can have wide-ranging regulatory effects is supported by the fact that long non-coding RNAs and related non-coding elements function as regulators or sponges of miRNAs, thereby indirectly influencing oncogene output and downstream signaling (Aalijahan and Ghorbian, 2019). Furthermore, the identification of co-regulated modules and the cell-type origins of expression changes has been made possible by transcriptomic analysis techniques like weighted gene co-expression network analysis (WGCNA) and careful deconvolution of bulk RNA-seq. These techniques have been successfully applied to uncover hub genes and pathway modules that connect transcriptomic shifts to phenotypic outcomes (Momeni et al., 2023a). The current study’s findings suggest that disrupting hsa-let-7c and hsa-miR-181c binding by rs712/rs9266 not only boost KRAS levels but might also alter local non-coding RNA-mRNA networks. It may activate downstream MAPK/ERK and PI3K/AKT pathways that cause proliferation and treatment resistance, which can be tested by combined transcriptome and proteomic profiling (Momeni et al., 2023a).

Overall, this study highlights the critical role of KRAS rs712 and rs9266 variants in BC development as well as progression by altering miRNA-mediated regulation. The downregulation of tumor-suppressive miRNAs hsa-let-7c and hsa-miR-181c in altered genotypes further supports their involvement in BC pathogenesis. These insights pave the way for future investigations into KRAS-targeted therapies and miRNA-based interventions to improve BC patient outcomes.

## Conclusion

This study highlights the significant role of rs712 and rs9266 within the 3′UTR of the KRAS gene in the BC gene in the development of BC. rs9266 disrupted miRNA-mediated regulation of KRAS, particularly by hsa-miR-181c, leading to increased KRAS expression. While rs9266 also affected hsa-let-7c binding, its impact was less significant. KRAS overexpression was observed in HT genotypes followed by HM genotypes in comparison with WT genotypes of both variants. The inverse correlation between KRAS expression and tumor-suppressor miRNAs, hsa-miR-181c and hsa-let-7c, highlights their regulatory roles in oncogenesis. Bioinformatics analysis revealed structural alterations in the KRAS 3′UTR due to genetic variations that might affect miRNA binding and regulatory dynamics. The inverse correlation between KRAS expression and tumor-suppressive miRNAs observed in RNA-seq data further supports their role in BC progression. Kaplan-Meier survival and ROC analyses reinforced the prognostic significance of KRAS, hsa-let-7c, and hsa-miR-181c, along with the association of overexpressed KRAS with poor outcomes, while elevated hsa-let-7c and hsa-miR-181c levels correlated with better OS. These findings establish KRAS and its regulatory miRNAs as potential diagnostic, prognostic, and therapeutic biomarkers.

### Strength

The current study exhibits several notable strengths. It employs an integrated multimodal design that combines genetic screening, functional validation, and expression analysis, thereby providing a comprehensive understanding of the molecular mechanisms involved. The use of a luciferase reporter assay to confirm miRNA–3′UTR interactions provide direct mechanistic evidence, reinforcing the credibility of the findings. Furthermore, the cohesive expression patterns observed across qRT-PCR, IHC, and Western blot analyses enhance the authenticity and reliability of the results. The application of multiple survival analysis tools, including KM Plotter and ROCplot, further strengthens the translational relevance of the study by linking molecular alterations to clinical outcomes. Importantly, the focus on tumor-suppressive miRNAs such as let-7c and miR-181c not only highlights their prognostic significance but also draw attention towards their potential as therapeutic targets in cancer research.

### Limitation

- A direct correlation between the observed variants in KRAS and BC risk could not be established due to the lack of a healthy control group. There was not much difference in the frequency of variants, when compared to data from the Indigenome population. India is a land of diversity in terms of housing of different ethnic groups. The controls therefore should have been from the same ethnic group to which the patients belonged to. This remains to be authenticated in the future study. Functional evaluation shows altered KRAS expression and miRNA binding confirming the role of KRAS in BC tumorigenesis.
- The generalizability of the expression results is limited by the relatively small biopsy sample size (n = 13). The consistency of the RT-PCR, Western blot, and IHC results support the findings. Validating these results in larger patient samples will be taken up in future.
- In order to establish a direct mechanistic link between the identified variants and downstream tumorigenic signaling, the impact of KRAS overexpression on key oncogenic signaling cascades, including MAPK/ERK and PI3K/AKT pathways needs to be understood by specific *in-vitro* and *in-vivo* experiments.
- Studies in other ethnic groups are warranted to figure out the applicability of these results in broader populations.

### Future directions

A two-pronged follow-up approach that combines focused mechanistic investigations with advanced computational probing of transcriptomes, building on the current functional and expression data needs to incorporated in future study. This will help to determine the contribution of various cell populations to the observed KRAS/miRNA expression patterns; identify co-expression modules and upstream regulators altered in variant carriers, and identify candidate lncRNAs or competing endogenous RNAs (ceRNAs) that interact with the let-7/miR-181 family. It will be helpful to first apply bulk RNA-seq deconvolution and WGCNA on larger cohorts (or reanalysis of public datasets) (Momeni et al., 2023b). Recent studies show the value of ML/AI and LLM-assisted approaches for biomarker discovery and diagnostic model building in oncology and BC specifically (Ghorbian and Ghorbian, 2023). By utilizing contemporary computational tools and machine-learning pipelines, predictive biomarker development and stratification of therapy response can be improved. Experimentally, it is possible to determine whether these 3′UTR variants cause prolonged activation of MAPK/ERK and PI3K/AKT signaling and change drug sensitivity by engineering the rs712/rs9266 alleles in pertinent BC cell lines using CRISPR/Cas9, along with miRNA perturbation (mimics/inhibitors) and downstream phospho-proteomic readouts. A systems-level understanding of how 3′UTR polymorphisms influence oncogenic networks and guide translational efforts for KRAS-driven BC is achievable through the integration of experimental data with computationally generated modules (Momeni et al., 2023a).

The current research provides mechanistic support that the pathophysiology of BC is associated with post-transcriptional deregulation of KRAS. To maintain physiological control over cell proliferation and survival signaling, tumor-suppressive miRNAs like hsa-let-7c and hsa-miR-181c normally attach to complementary locations within the 3′UTR of KRAS gene and inhibit its translation. However, these miRNA binding sites are disrupted by the reported variations like rs712 and rs9266, which result in decreased repression and KRAS overexpression. According to earlier research, constitutive activation of the MAPK/ERK and PI3K/AKT pathways is driven by elevated KRAS activity, that promotes oncogenic transformation, invasion, and treatment resistance in some cancers (Lam et al., 2022; Chhichholiya et al., 2024).

This work offers fundamental proof that polymorphisms in the KRAS gene’s 3′UTR (rs712 and rs9266) alter post-transcriptional regulation by tumor-suppressive miRNAs, which in turn aids in the progression of BC. Building on these results, a few directions are worth investigating further. In order to overcome the ethnic constraint of the current cohort, extensive, multi-ethnic association studies should be carried out to confirm the prevalence and clinical significance of these KRAS mutations across a range of populations. Second, to clearly define the downstream effects of these variations on the MAPK/ERK and PI3K/AKT signaling cascades, functional studies using *in-vitro* (CRISPR/Cas9-based editing, miRNA-mimic or inhibitor tests) and *in-vivo* BC models are necessary. These studies will provide light on the relationship between changed miRNA binding and persistent oncogenic activity. Third, transcriptomic and proteomic characterization of cells harboring variants may reveal additional miRNA–mRNA interactions disrupted in carcinogenesis and reveal larger regulatory networks impacted by KRAS dysregulation. Lastly, as new precision-medicine approaches, the possibility of reestablishing miRNA-mediated repression by miRNA-based therapies, antisense oligonucleotides, or small-molecule modulators that target KRAS stability should be investigated. When combined, these approaches will increase the current findings’ translational significance and might speed up the creation of therapeutic and prognostic measures for breast tumors driven by KRAS.

## Data Availability

Link for Sequencing data (KRAS variant ras712 & rs9266)- https://drive.google.com/drive/folders/1GWlVrSIewubbjqxAAJn1D5aWct1nzoYH?usp=sharing. The platform for KM plot analysis is- https://kmplot.com/analysis/index.php?p=service; https://kmplot.com/analysis/index.php?p=view&pa_id=27257706&show=bZJNbsQwCIXvknVVaRbd9DKIOHiC4hgLcKY_6t3rZDGTZGb7eH58Bn47w4UADZxj7D67iMmoe-tslBvMNDDmprrWh8h5xq-T2BPZDXU-JxSVIuosGdDBqi68YDq7egxTez2AUiSlHOg1CUjcZ-wBShKHxJnARw5TJrPmuBzKTl8OvaThdcX4Z-17ef841EI1lxlm5NyG5OmJbXOVhlQJihivv13ppChfRz8S0pXysLf14i0-Ubwbc5170nVeyjbtuo08EATKJsr52mDCZGeYIEkUCiZy35Ouiwgjhekxt7sEmNINv_dZ1QgWVMY-EcSWGGRse9xbgqiCFWpb327koK-ySX6iqy7bmW0If_8 and for ROC analysis is https://rocplot.com/site/treatment.
