# Supplementary material for: Evaluation of genetic variation in tumor suppressor miRNA encoding and their target genes in breast cancer; focus on miRNA interaction and expression analysis
Source: Front Genome Ed. 2026 Feb 27;8:1705463. doi: 10.3389/fgeed.2026.1705463 (PMC12982462; doi:10.3389/fgeed.2026.1705463)
Supplement: Supplementary file 1 [file Supplementaryfile1.docx]

**Supplementary Data 1**

**Table 1: Primers for *hsa-let-7c*, *hsa-miR-34a*, *hsa-miR-145a* encoding gene and 3’UTR of *KRAS, IGFBP6* and *IGF1R* genes and the annealing temperature**

| **Gene** | **Primer** | **Annealing Temperature** |
| --- | --- | --- |
| *hsa-Let-7c* | Forward: 5'-CGTGACCTATGCTGGAAACCC-3'  Reverse: 5'-TACTACCTCAACCCGGATGC-3' | 58º C |
| *hsa-miR-34a* | Forward: 5'-AGGCCTTCCTGCATAGTAAGTG-3'  Reverse: 5'-CATCCTTTCTTTCCTCCCCACA-3' | 58º C |
| *hsa-miR-145a* | Forward: 5'-CAGAGGGTTTCCGGTACTTT-3'  Reverse: 5'-AGCCTCACAGGGATGTTATGAAA-3' | 58º C |
| *KRAS* | Forward: 5'-GCTCCATGCAGACTGTTAGC-3'  Reverse: 5'-CTGGGTCTGCCTTAACAGGA-3' | 58º C |
| *IGFBP6* | Forward: 5'-TGGCTCTGCCTGATTTCTGA-3'  Reverse: 5'-ATAGTCTCACCTGCCGCTTC-3' | 56º C |
| *IGF1R* | Forward: 5'-CACAAGCCTCCTGTACCTCA-3'  Reverse: 5'-CTGGAAACCCCTTCCCATCT-3' | 60º C |

**Table 2. Reaction steps used in the thermal cycle for cDNA synthesis**

| S.No. | Reaction Step | Time |
| --- | --- | --- |
| 1. | 25ºC | 5 minutes |
| 2. | 46ºC | 20 minutes |
| 3. | 95ºC | 4 minutes |
| 4. | 4ºC | ∞ |

**Table 3: The sequence for the cDNA-specific primer used for enrichment of miRNA**

| **cDNA-specific Primer** | **Sequence** |
| --- | --- |
| Let-7c-3p | 5'-GTCGTATCCAGTGCAGGGTCCGAGGTATTCGCACTGGATACGACGGAAAG-3' |
| miR-181c-5p | 5'-GTCGTATCCAGTGCAGGGTCCGAGGTATTCGCACTGGATACGACACTCAC-3' |

**Table 4: Primers used for qRT-PCR**

| **S. no.** | **Gene name** | **Primers** | **Annealing Temperature** |
| --- | --- | --- | --- |
| 1. | *hsa-let-7c* | Forward: 5'-AACCGGCTGTACAACCTTCT-3′  Reverse: 5'-GTCGTATCCAGTGCAGGGT-3' | 53º C |
| 2. | *hsa-miR-181c* | Forward: 5'-AACATTCAACCTGTCGGTGAGT-3'  Reverse: 5'-GTCGTATCCAGTGCAGGGT-3' | 62.5º C |
| 3. | *U6 snRNA* | Forward: 5′-CTCGCTTCGGCAGCACA-3′  Reverse: 5′-AACTCTTCACTAATTTGCGT-3′ | 56º C |
| 4. | *KRAS* | Forward: 5′-TGAGGACTGGGGAGGGCTTT-3′  Reverse: 5′-AGGCATCATCAACACCCTGTCT-3′ | 61º C |
| 5. | *β-actin* | Forward: 5′-AGAGCTACGAGCTGCCTGAC-3′  Reverse: 5′-AGCACTGTGTTGGCGTACAG-3′ | 60º C |

**Table 5: Reaction mixture used in the qRT-PCR reaction setup**

| **Reactants** | **Volumes** |
| --- | --- |
| **cDNA** | 4.5 μl |
| **Forward Primer** | 0.5 μl |
| **Reverse Primer** | 0.5 μl |
| **SYBR-Green** | 5 μl |

**Table 6: Reaction conditions for RT-PCR**

| **Process** | **Temperature** | **Time of cycles** | |
| --- | --- | --- | --- |
| Initial Denaturation | 94ºC | 3 minutes | |
| Denaturation | 94ºC | 45 seconds | 40X |
| Annealing | 57ºC - 62ºC | 30 seconds |  |
| Extension | 72ºC | 45 seconds |  |
| Final Extension | 72ºC | 2 seconds | |
|  | 4ºC | ∞ | |

**Table 7: Demographic details of the BC patients**

| **Demographic/Clinical details** | **No. of Patients** |
| --- | --- |
| **Age at diagnosis**  <=45 year  >45 year | n=59 (28.36 %)  n=149 (71.63 %) |
| **Age at 1^st^ pregnancy**  >=25 year  <25 year  5 Patients Unmarried | n=119 (58.62 %)  n=84 (41.37 %) |
| **Menopausal status**  Pre-menopausal  Post-menopausal | n=91 (43.75 %)  n=117 (56.25 %) |
| **Tumor Grade**  Grade 2  Grade 3 | n=170 (81.73 %)  n=38 (18.27 %) |

**Table 8: Distribution of rs712 & rs9266 in the 3’UTR of *KRAS* gene, genotypic and allelic frequencies**

| ***KRAS* Gene**  **(rs712)** | **Genotype** | **Genotypic frequency** | **Allele** | **Allelic Frequency** |
| --- | --- | --- | --- | --- |
|  | **TT** | 27 (12.98 %) | **T** | 0.3846 |
|  | **TG** | 106 (50.96 %) |  |  |
|  | **GG** | 75 (36.05 %) | **G** | 0.6154 |
|  | Combined | |  |  |
|  | **TG/GG** | 181 (87.01%) |  |  |
| ***KRAS* Gene**  **(rs9266)** | **Genotype** | **Patient** | **Allele** | **Allelic Frequency** |
|  | **TT** | 27 (12.98 %) | **T** | 0.3846 |
|  | **TC** | 106 (50.96 %) |  |  |
|  | **CC** | 75 (36.05 %) | **C** | 0.6154 |
|  | Combined | |  |  |
|  | **TC/CC** | 181 (87.01%) |  |  |
