# Supplementary material for: Evaluation of genetic variation in tumor suppressor miRNA encoding and their target genes in breast cancer; focus on miRNA interaction and expression analysis
Source: Front Genome Ed. 2026 Feb 27;8:1705463. doi: 10.3389/fgeed.2026.1705463 (PMC12982462; doi:10.3389/fgeed.2026.1705463)
Supplement: Supplementary file 2 [file Supplementaryfile2.docx]

**Supplementary Data 2**


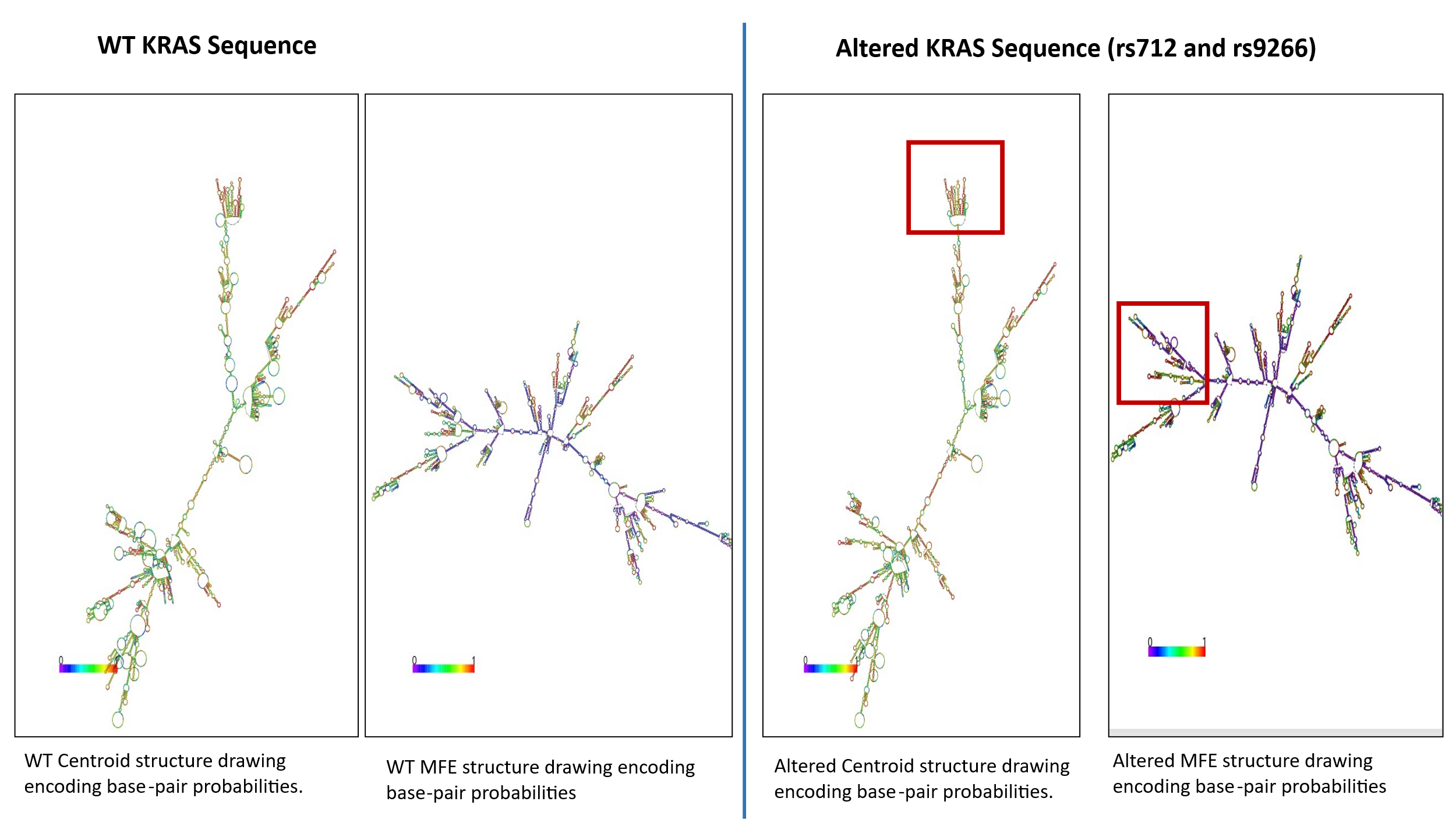


**Figure 1:** Optimal RNA secondary structure for WT vs. altered *KRAS* sequence. It presents the optimal secondary structures for both the wild-type and mutant *KRAS* genes, along with the centroid and MFE representations. The base-pair probabilities are encoded with darker colors representing more stable base-pairing.


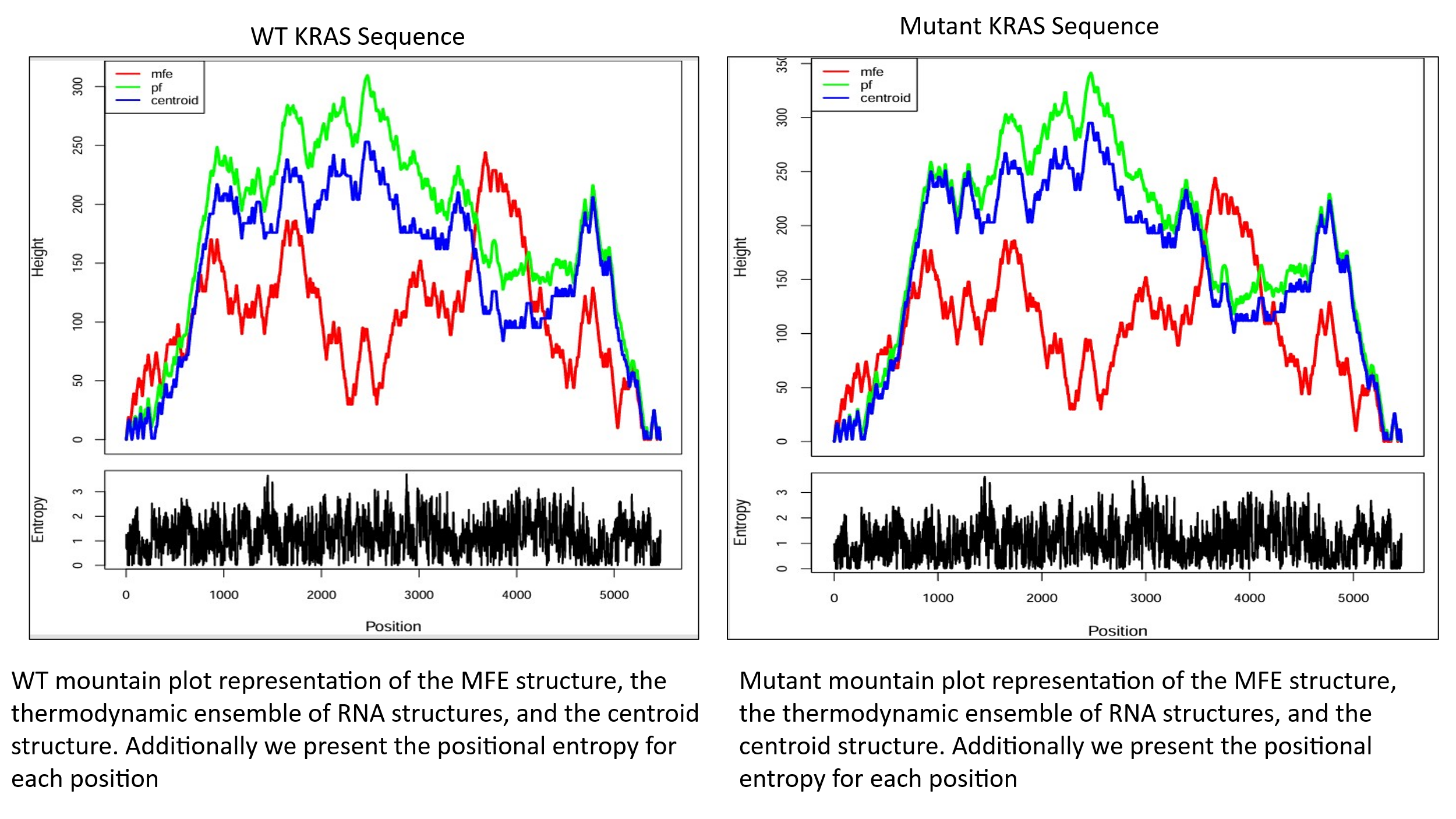


**Figure 2:** Mountain Plot of WT vs. altered *KRAS* Sequence


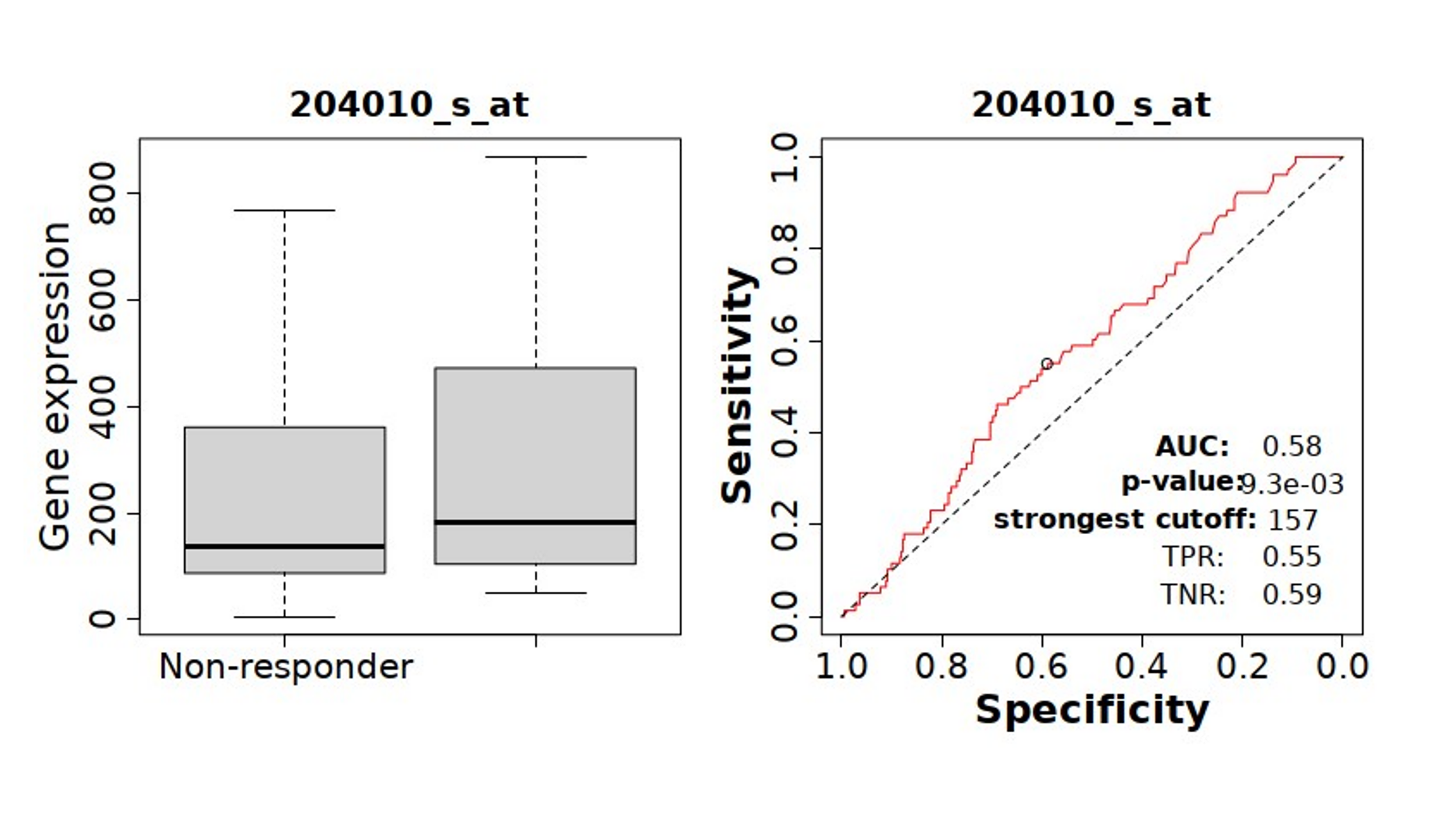
**Figure 3**: ROC Plot for *KRAS* Expression in grade 2 BC patients receiving chemotherapy

**
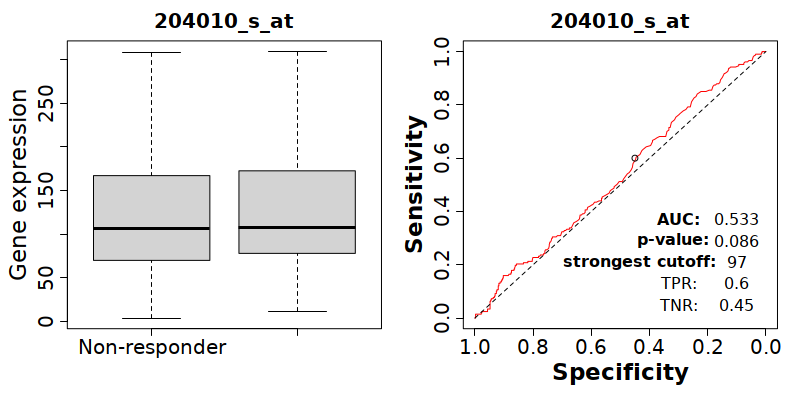
**

**Figure 4:** ROC Plot for *KRAS* Expression in grade 3 BC patients receiving chemotherapy


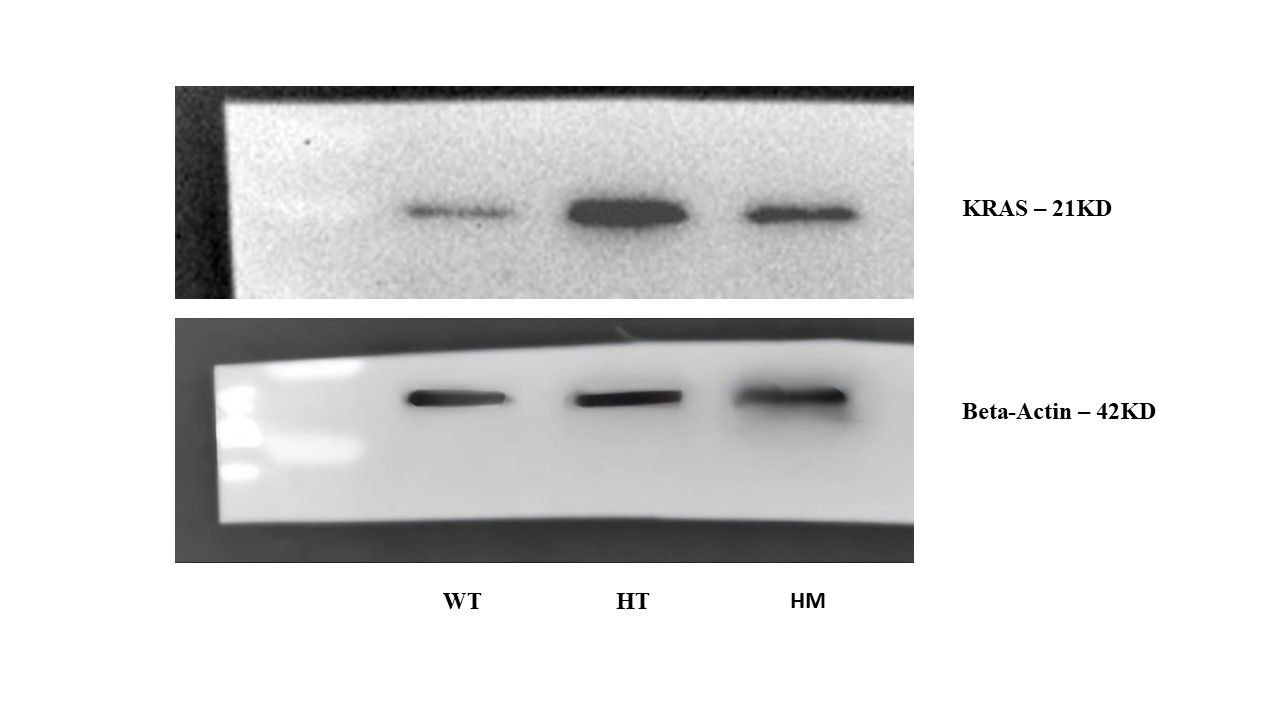


**Figure 5:** (A) Western Blot Analysis (original gel) of *KRAS* in WT genotype rs712 (TT) & rs9266 (TT); HT genotype rs712 (TG) & rs9266 (TC) and HM genotype rs712 (GG) & rs9266 (CC). The control image shown here has not been used before for illustration.
